# Comparison of Functional Outcome and Quality of Life in Patients With Idiopathic Scoliosis Treated by Spinal Fusion

**DOI:** 10.1097/MD.0000000000003289

**Published:** 2016-05-13

**Authors:** Hengwei Fan, Qifei Wang, Zifang Huang, Wenyuan Sui, Jingfan Yang, Yaolong Deng, Junlin Yang

**Affiliations:** From the 1st Affiliated Hospital of Sun Yat-sen University, Zhongshan Er Road, Guangzhou, China.

## Abstract

Longer spinal fusions have been shown to result in improved deformity correction; however, loss of normal flexibility in the fusion area should not be ignored. Current consensus was to achieve a shorter fusion in primary surgery, with the goal of preserving as much of the distal motion segment as possible. However, the correlation between the length of fusion and functional outcome remains controversial. To the best of our knowledge, a previous study has demonstrated the function outcomes and the differences in HRQoL with specific fusion levels.

In this cross-sectional study, 172 patients (mean age, 17.8 y) with idiopathic scoliosis treated by spinal fusion (mean time since surgery, 29.7 mo) were included to measure lumbar spine mobility and quality of life using validated outcome instruments in the study population. Patients were assigned to 5 groups according to the lower instrumented vertebra (LIV) level: group A (fusion above L2) 26 patients; group B (fusion to L2) 21 patients; group C (fusion to L3) 46 patients; group D (fusion to L4) 53 patients; and group E (fusion to L5) 26 patients. At each follow-up, patients were asked to complete the Scoliosis Research Society 22 (SRS-22) Questionnaire. Lumbar mobility was assessed using a dual digital inclinometer.

Average spinal range of motion (ROM) was 41.4 degrees (SD, 20.7), forward flexion was 29.2 degrees (SD, 15.0), and backward extension was 12.2 degrees (SD, 9.5). The total spinal range of motion and forward flexion dropped noticeably as the LIV got more distal. Statistically significant between-group differences (1-way ANOVA) were found for ROM (*P* < 0.001), forward flexion (*P* < 0.001), or backward extension (*P* < 0.001). The motion segments preserved significantly correlated with ROM (*r* = 0.76, *P* < 0.001), ROMF (*r* = 0.76, *P* < 0.001), and ROME (*r* = 0.39, *P* < 0.001). However, no significant between-group differences was found for each domain of SRS-22 questionnaire.

The motion segments preserved strongly correlated with lumbar mobility. Less fusion levels can preserve better lumbar flexibility by keeping more motion segments.

## INTRODUCTION

All pedicle screw posterior instrumentation with spinal fusion is the main surgical procedures in idiopathic scoliosis (IS) patients aiming to achieve a balanced spine and to improve trunk deformity.^1–4^ Although longer spinal fusions have been shown to result in improved deformity correction, loss of normal flexibility in the fusion area should not be ignored.^5–7^ Junctional hypermobility caused by loss of spinal motion over time may contribute to back pain due to accelerated lumbar degeneration and may necessitate a more extensive fusion in adulthood.^8,9^ This has resulted in current consensus to achieve a shorter fusion in primary surgery, with the goal of preserving as much of the distal motion segments as possible. However, the reverse correlation between the length of fusion and functional outcome has not been conclusively demonstrated. Several studies have shown that patients with general longer spinal fusions has resulted in a greater functional loss and a higher incidence of back pain.^10,11^ To the best of our knowledge, no previous study has demonstrated the outcome of function, and the differences in health-related quality of life (HRQoL) with specific fusion levels. The purposes of this study are to evaluate and compare the clinical, radiographic, and functional outcome of spinal fusions terminating at different levels in IS patients. Besides, the Scoliosis Research Society 22 (SRS-22) questionnaire was applied to determine the HRQoL of IS. Our hypotheses are that less fusion levels can preserve better lumbar flexibility by keeping more motion segments, and can improve patients’ HRQoL.

## MATERIALS AND METHODS

### Study Design

This cross-sectional study was approved by the ethics committee of our hospital. Consecutive patients with IS who had undergone posterior spinal fusion with all pedicle screws instrumentation were recruited with informed consent. Patients who needed revision surgery for implant loosening or pseudoarthrosis were excluded. All patients were divided according to their lowest instrumented vertebras (LIVs). Group A consists of patients who had undergone a thoracic fusion with the LIV at L1 or above. Group B, C, D, or E comprises of patients who had undergone fusion with the LIV at L2, L3, L4, or L5, respectively.

### Range of Motion (ROM) Measurement

T12 and L5 were located by surface bony landmarks (the posterior superior iliac spine and the inferior angle of scapula) with the patients in the prone position. Then, the T12–L1 and L5–S1 interspinous spaces were marked on the skin. After a brief warmup procedure (including trunk flexion, extension, lateral flexion, and axial rotation on both sides), the ROM of the lumbar spine was performed with an dual digital inclinometer (6 ROM Microfet, Hoggan, UT) with 2-point contact at its base for which the application in IS patients have been described.^12^ The device can be easily used in daily practice. The participant stood against a low wall in relaxed posture with feet about shoulder width apart to restrain the movement of the pelvis. After that, the inclinometer was placed on premarked spots and calibrated again to zero. Thereafter, the participant was asked to bend forward and then backward to the maximal level. The readings at T12–L1 and L5–S1 were recorded, and the range of flexion motion (ROMF) and the range of extension motion (ROME) were calculated by subtracting the measurement at L5–S1 (reflecting the pelvic movement) from the measurement at T12–L1 (reflecting both lumbar and pelvic movement) gives the regional lumbar motion. The total range of motion (ROM) was the sum of the ROMF and ROME.

### SRS-22 Questionnaire

Scoliosis-specific questionnaire was selected to evaluate the HRQoL of patients after the spinal fusion treatment. Patients were assessed preoperatively and at the latest follow-up. A simplified Chinese version SRS-22 questionnaire^13^ was applied in our study. The questionnaire covers 5 domains (function/activity, pain, self-image, mental health, and satisfaction with treatment). Each item has 5 verbal response alternatives ranging from 1 (worst) to 5 (best). Results are expressed as the mean (total sum of the domain divided by the number of items answered) for each domain.

### Statistical Analysis

All continuous variables were presented as mean ± SD for each group and were subjected to statistical analysis. The normality test was conducted to examine the shape and distribution of the continuous variables. One-way ANOVA or Kruskal–Wallis nonparametric test was used to compare differences between groups, and Bonferroni post hoc testing was used to identify specific group interactions. For the bivariate analyses, the Pearson correlation coefficient was calculated. The statistical analysis was performed using SPSS (version 12, Chicago, IL). All the tests were 2-tailed and the significance level (*P* < 0.05) were considered statistically significant.

## RESULTS

### Clinical and Radiologic Findings

The study included 172 patients (128 females, 44 males), with a mean age of 17.8 years (range 9–26 y) at enrollment. Mean age at the time of surgery was 15.3 years (range 7–20 y). All patients had been undergone posterior spinal fusion and instrumentation. The LIV was above L2 in 26 case, L2 in 21, L3 in 46, L4 in 53, L5 in 26. The mean interval between surgery and the study follow-up visit was 29.7 months (range 24–144 mo). The radiologic magnitude of the major curve was 63.2 degrees (range 31.2–131.8 degrees) preoperatively and 15.0 degrees (range 1.5–54.1°) at last follow-up. Detailed data were provided in Table 1.

**TABLE 1:**
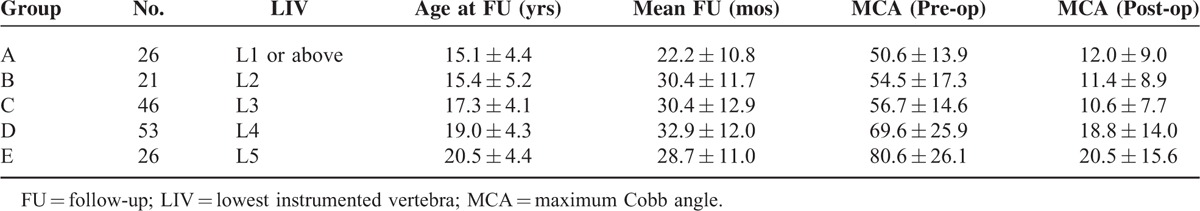
Clinical Data for Patients With AIS Undergoing Surgery

### Spinal Range of Motion

**FIGURE 1:**
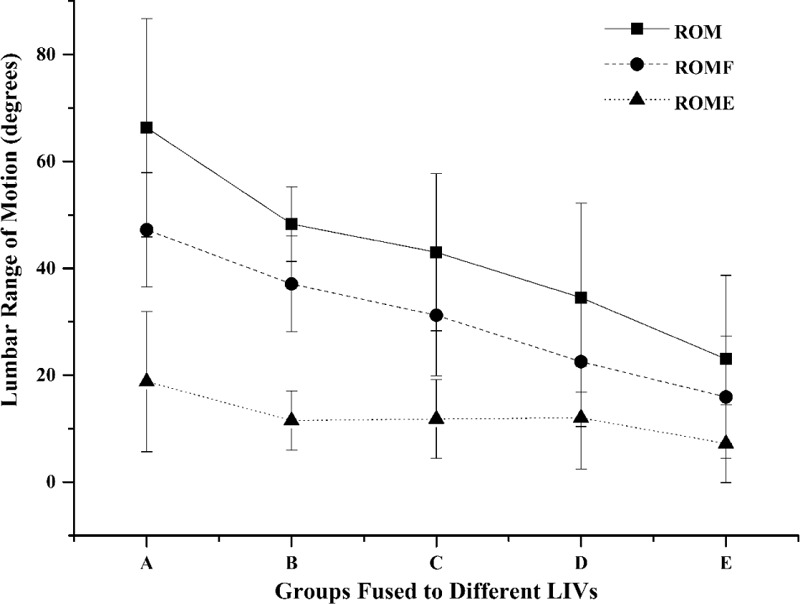
ROM of the fused spine in different groups. One-way ANOVA test showed significant differences in ROM (*P* < 0.001), ROMF (*P* < 0.001), and ROME (*P* < 0.001).ROM = range of motion, ROME = range of extension motion, ROMF = range of flexion motion.

Average spinal ROM was 41.4° (SD, 20.7), forward flexion was 29.2° (SD, 15.0), and backward extension was 12.2° (SD, 9.5). Mobility according to the LIV group is shown in Figure 1 and Table 2. The total spinal range of motion and forward flexion dropped noticeably as the LIV got more distal. The main reason lead to the reduction in ROM, ROMF, and ROME could be the fusion of L1/2 disc and L4/5 disc. Statistically significant between-group differences (1-way ANOVA) were found for ROM (*P* < 0.001), forward flexion (*P* < 0.001), or backward extension (*P* < 0.001). Post hoc analysis demonstrated no significant differences between groups B and C, C and D in total ROM, and no significant differences between groups B and C, D and E in ROMF. As for ROME, significant differences were found between group A and groups C, D, E.

**TABLE 2:**
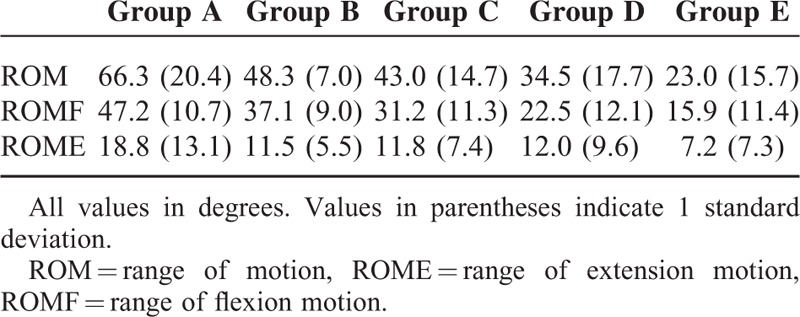
ROM of the Fused Spine in Different Groups

Neither the age nor follow-up interval correlated with the spinal range of motion. The motion segments preserved significantly correlated with ROM (*r* = 0.62, *P* < 0.001), ROMF (*r* = 0.66, *P* < 0.001), and ROME (*r* = 0.30, *P* < 0.001). Significant inverse correlations were observed between the maximum Cobb angle before surgery or at latest follow-up and ROM or ROMF (Table 3).

**TABLE 3:**
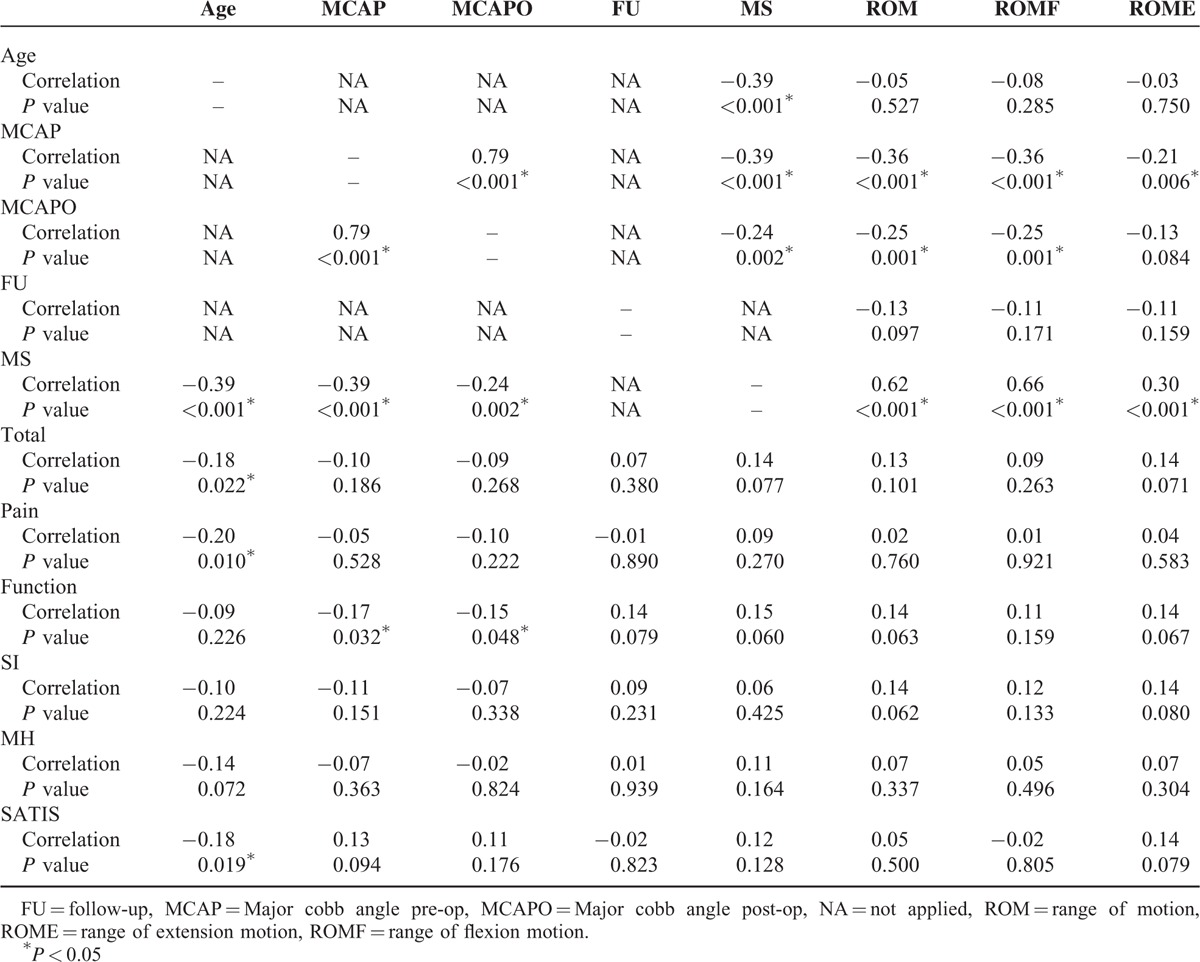
Correlations Between Spinal Range of Motion and the Radiographic Measurements, SRS-22 Scores, and Related Parameters

### Health-Related Quality of Life

**FIGURE 2:**
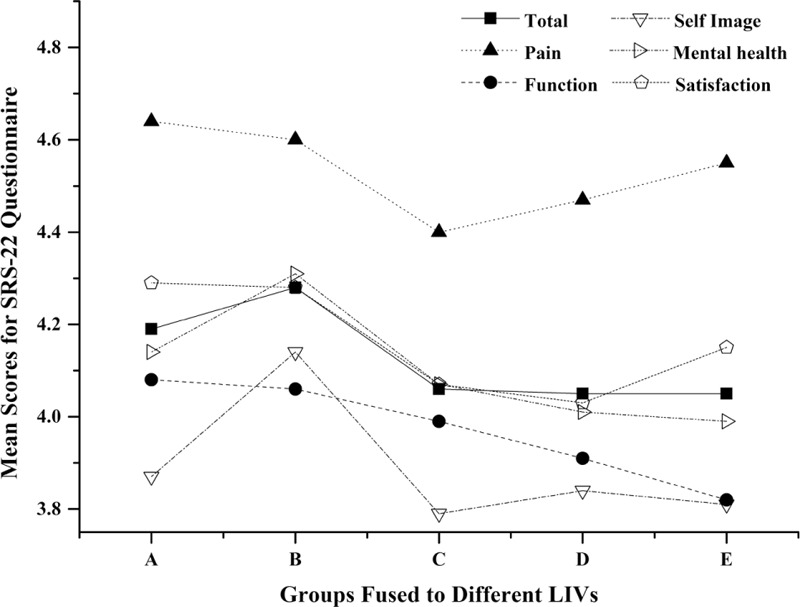
Mean scores of the each domain of SRS-22 questionnaire in different groups.

Quality of life was assessed with the SRS-22 Questionnaire. The various subscales yielded the following scores: function 4.0 (SD, 0.6), pain 4.5 (SD, 0.5), body image 3.9 (SD, 0.6), mental health 4.1 (SD, 0.6), and satisfaction 4.1 (SD, 0.7); the mean total score was 4.1 (SD, 0.4). SRS-22 Outcome Score Means according to the LIV group was shown in Figure 2. No significant between-group differences (1-way ANOVA) were found for each domain. However, a descending trend was observed for function domain as the LIV got more distal. No significant correlations were observed between the SRS-22 function domain and the follow-up duration (*r* = 0.14, *P* = 0.079), motion segments remained (*r* = 0.15, *P* = 0.060), ROM (*r* = 0.14, *P* = 0.063), ROMF (*r* = 0.11, *P* = 0.159), and ROME (*r* = 0.14, *P* = 0.067) (Table 3).

## DISCUSSION

As well as achieving a balanced spinal fusion to keep scoliosis from progressing, preserving motion segments has always been a desirable goal. A loss of spinal mobility could be detected following extensive vertebral fusion, and scoliosis patients with spinal fusion have been proven to have less spinal mobility than untreated scoliosis patients and healthy controls.^5–7,14,15^ In this study, lumbar mobility was measured with a dual digital inclinometer, for which the parameters of validity have been described. The lumbar mobility values we found are similar to those reported by other researchers using different devices.^16,17^ Aaro and Ohlen^18^ found gradual loss of flexion of the lumbar spine as the distal end of the fusion moved from T12 to L4, using a similar measurement technique. They reported patients fused to T12 preserved a good lumbar flexibility as do normal people and a gradual drop of ROMF from 67° to 23° as the LIV got more distal. They concluded that the residual lumbar mobility after extensive spinal fusion is largely determined by the number of fused segments. Our results supported this idea and coincide with other studies in which there are linear correlation between the range of motion in the lumbar region and the number of preserved motion segments.^6,7,11,15,18–20^ With the fusion level extending to the low lumbar spine, mobility was less than in those with fusion including only the more cephalad lumbar vertebrae.

However, no differences in ROM was found between patients fused at L2 and those at L3, or patients fused at L3 and those at L4, nor was there any difference in ROMF between patients with LIV at L2 and L3, or patients fused at L4 and those at L5. In the study of Sanchez-Raya,^12^ patients were assigned into 3 groups according to the LIV level (group 1: T12, L1, L2; group 2: L3; group 3: L4, L5, S1). The ROMF for each group was 41.4, 32.9 and 20.2°; however, significant difference was only found for group 3 relative to other groups. Meanwhile, results of the study by Winter and colleagues^21^ showed that functional spine motion was quite good with the exception of those patients fused at L4. According to lumbar spinal movements measured by x-ray analysis, L4/5 disc constitute the largest proportion of either the ROM or ROMF.^22^ These insignificant decrease mentioned above could be explained by the relative less contribution to lumbar function or the small sample size. The reason, we speculate, may also have something to do with the patient's previous mobility. However, the importance of the preoperative lumbar mobility was unable for evaluation because this data was unavailable.

The loss of ROM resulting from spinal fusion might lead to low back pain, trunk rigidity, and thus could have a negative impact on health-related quality of life.^23,24^ The SRS-22 questionnaire is a patient-reported outcome instruments that has proven to be validated for the AIS population.^25,26^ In our study, patients scored around 4 points in all domains which implies that these patients were satisfied. Results revealed that no significant between-group differences was found for each domain despite of a descending trend. There have been several studies demonstrating that no differences was found for patients with different LIVs which coincided with the current one. Green et al^27^ could not show any difference for subpopulations of patients with lowest instrumented vertebra of T12 or L1 and L3 as measured by SRS-22 scores. Similar findings were also described by Ding et al^28^ that no significant difference could be identified between different fusion levels. However, in 2012, Sanchez-Raya and colleagues^12^ reported statistical difference in terms of the SRS subtotal score and the pain subscale between patients fused distal to L3 and above. Our data also show that the patient's subjective impression of spine flexibility measured by SRS-22 function domain could reflect the loss of movement after vertebral fusion. But no significant correlation was found between ROM and the SRS subtotal or the function subscore. Similar results were reported using the mean score of perception of TF measured by QLPSD instrument between the LIV groups, and the correlation between TF and lumbar flexion was not significant.^12^ We speculate that the SRS-22 questionnaire may not be sensitive enough at least for the function domain. And the relatively low Pearson correlation coefficients could indicate that scores of function domain is conditioned by other factors as well. By including more variables, we noted that no association was detected between follow-up duration and function subscore which long-term lumbar function may not affected by extensive blunt dissection of paraspinal muscles, postoperative scarring, or rehabilitation exercise. The sagittal curves (cervical lordosis, thoracic kyphosis, and lumbar lordosis) and spinal-pelvic parameters have been correlated with quality of life in patients with scoliosis;^29–31^ however, the lack of these radiographic data was an important limitation in the present study.

In conclusion, lumbar ROM and ROMF showed strong correlation with LIV, suggesting that the number of preserved motion segments can have an influence on the residual mobility. Data indicated that the L1/2 and L4/5 are the most important motion segments that affect. But our results failed to correlate the follow-up duration and the residual mobility, which mean the ROM of lumbar spine may not improve with time. The patients did had a positive perception of their activity function; however, no significant between-group differences was found for SRS-22 function domain despite of a descending trend. This could suggest that the function domain score may be influenced by other factors and may not be sensitive enough. Functional outcome of spinal fusions terminating at different levels in our IS patients justify the need of saving fusion levels while achieve solid fusion in a well-balanced spine.
